# Analysis of physiological changes in peanut germination and seedling stages following low-temperature stress

**DOI:** 10.3389/fpls.2026.1787958

**Published:** 2026-03-27

**Authors:** Zhihao Xiong, Shutao Yu, Zhuo Gao, Shengnan Xu, TengJiao Wang, Yechao Yin, Yu Zhang, Jingchao Dong, Xueying Li

**Affiliations:** 1 Liaoning Desertification Control and Utilization Research Institute, Fuxin, China; 2 Faculty of Agronomy Jilin Agricultural University, Changchun, China

**Keywords:** enzyme activity, germination stage, low temperature, peanut, seedling stage

## Abstract

**Introduction:**

Peanut cultivation in high-latitude regions is frequently subjected to low-temperature stress, which adversely affects peanut growth and development. The correlation between physiological changes in peanut during the germination and seedling stages and exposure to low-temperature stress at the harvest period remains unclear, and this research gap limits the in-depth study of peanut cold tolerance. This study aimed to explore the above correlation and identify core physiological indicators for evaluating peanut cold tolerance at germination and seedling stages.

**Methods:**

Thirty-six peanut accessions were used as experimental materials and subjected to natural low-temperature stress during the field harvest period. After drying the pods for 3 days, laboratory germination tests were conducted to screen out one extremely cold-tolerant and one extremely cold-sensitive accession from the 36 materials. The two extreme materials were then exposed to artificial low-temperature stress at the germination and seedling stages: the temperature was decreased from 26 °C to target temperatures (8 °C, 4 °C, and 0 °C) at a rate of 2 °C/h, and then increased back to 26 °C at the same rate, with a control group maintained at a constant 26 °C. Physiological indicators of the materials were determined at five time points after they returned to 26 °C and resumed normal growth, and principal component analysis (PCA) was used for comprehensive analysis of the indicators.

**Results:**

After low-temperature stress at both germination and seedling stages, the cold-tolerant material had significantly higher contents of proline (Pro) and soluble sugar, as well as higher activities of Superoxide Dismutase (SOD), Peroxidase (POD), and Catalase (CAT) compared with the cold-sensitive material, while its Malondialdehyde (MDA) content was significantly lower. All physiological indicators of the cold-tolerant material recovered to normal levels within 36 hours of recovery, whereas the recovery of the cold-sensitive material was significantly slower. PCA extracted three principal components from the measured indicators, with a cumulative contribution rate of 89.35%. Pro and SOD were ultimately identified as the core indicators for evaluating peanut cold tolerance at the germination and seedling stages.

**Discussion:**

This study successfully screened two peanut materials with extreme cold tolerance phenotypes by combining field natural low-temperature stress and laboratory simulated low-temperature stress treatments. The clarification of Pro and SOD as core evaluation indicators fills the research gap in the cross-stage cold tolerance study of peanuts, which links harvest-period low-temperature stress with physiological responses at germination and seedling stages. The screened extreme cold-tolerant and cold-sensitive materials provide important germplasm resources for subsequent peanut cold tolerance breeding, and the identified core indicators lay a solid theoretical foundation for the rapid evaluation and identification of peanut cold tolerance.

## 1 Introduction

Peanut *(Arachis hypogaea* L.*)* is a globally cultivated economic crop (Zheng and Sun, 2024), serving as both a vital protein source and primary raw material for food processing (Zhuang and Chen, 2019; Zhang and Ma, 2022), making it a significant grain and oil crop worldwide. In recent years, with the increasing frequency of extreme weather globally, peanut germination and harvest in high-latitude regions are often constrained by low-temperature stress (Kim and Lee, 2015). Studies indicate that under severe low-temperature stress during the seedling stage, excessive reactive oxygen species (ROS) accumulation leads to cellular toxicity and cell death (Li and Zhang, 2014), ultimately inducing overall plant decline. Early frosts before autumn harvest directly impact pod filling and maturation, resulting in increased empty pods, reduced oil content, degraded quality, and severe yield losses (Chen and Cheng, 2020). Therefore, screening and breeding cold-tolerant peanut germplasm, along with in-depth investigation of peanut’s response mechanisms to low-temperature stress, holds urgent practical significance for developing stress-resistant varieties and addressing climate change challenges. Through extensive evolutionary adaptation, plants have developed regulatory mechanisms to cope with low-temperature stress (Nakaminami and Seki, 2018). Physiologically and biochemically, low-temperature stress induces a series of changes in plant cells (Zhang and Huang, 2015; Kumar et al., 2018). Free oxygen species attack unsaturated fatty acids within cell membranes, triggering lipid peroxidation and leading to increased Malondialdehyde (MDA) levels (Zhang and Huang, 2015; Ravi and Foyer, 2023). At this stage, soluble sugars and Pro can serve as osmoprotectants through active accumulation (Zuther and Buchel, 2004; Ma and Zhang, 2009; Dong and Lin, 2021), lowering the intracellular freezing point, preventing cellular dehydration, and thereby stabilizing cell structure (O’Hara and Paul, 2013). Additionally, they can act as scavengers for ROS generated by low-temperature stress (Zhang and Dong, 2019). Simultaneously Dismutase (SOD) serves as the first line of antioxidant defense (Saed-Moucheshi and Sohrabi, 2021), converting superoxide anion into H_2_O_2_ Peroxidase (POD) catalyzes the oxidation of H_2_O_2_ using various substrates (Sheteiwy and Ali, 2021), while Catalase (CAT) decomposes H_2_O_2_ into water and hydrogen gas (Wei and Xu, 2020). Furthermore, plants have evolved a series of complex network regulatory mechanisms to respond to cold stress, primarily including C-repeat Binding Factor (CBF)-dependent signaling pathways and non-CBF-dependent signaling pathways (Shi et al., 2018). Under cold stress, the transcription factor inducer of CBF Expression 1 (ICE1) binds to the promoter region of CBF genes, activating their expression. The activated CBFs further activate the expression of downstream Cold-Responsive genes, thereby enhancing plant cold tolerance (Qian et al., 2024). While numerous studies have examined cold stress during peanut germination and seedling stages (Rudrabhatla and Rajasekharan, 2002; Wang and Liu, 2021; Patel and Khandwal, 2022; Yang and Teng, 2023). However, current research on peanut cold tolerance primarily focuses on single growth stages (such as germination or seedling stages alone) and predominantly employs artificial low-temperature treatments on materials that have not undergone natural field stress.

This approach overlooks the critical role of the harvest period in yield formation and seed quality accumulation. Cold stress experienced during the harvest period can fundamentally alter seed vigor, triggering a series of physiological shifts that persist through germination and dictate the resilience of the emerging seedlings. This inter-stage cold tolerance linkage remains a significant research gap. The intrinsic relationship between harvest-period low-temperature stress and physiological changes during germination and seedling stages remains unclear. Furthermore, there is a lack of systematic studies screening extreme materials based on natural low-temperature stress during the field harvest period to investigate subsequent physiological mechanisms of cold tolerance.

This study addresses this core gap in existing research. The novelty of this study lies primarily in its innovative research perspective, which breaks through the limitations of single-stage studies by focusing on the cross-stage correlation between “focusing on the physiological continuity from initial cold stress at harvest through to the subsequent responses during germination and the recovery of seedlings.” For the first time, it investigates the patterns and intrinsic connections of changes in cold-tolerant physiological traits during the subsequent germination and seedling stages after peanuts encounter low-temperature stress at harvest. This research fills a gap in the study of cross-stage cold tolerance in peanuts. To address this research gap, this study utilized 36 self-bred high-oleic peanut germplasm resources provided by the Peanut Breeding Team at the Liaoning Institute of Sandland Management and Utilization. These materials, derived from diverse hybrid combinations, exhibit rich genetic diversity.

The study first subjected these 36 peanut materials to low-temperature stress in the field during harvest. After natural air-drying, germination tests were conducted. Based on indicators such as germination rate, sprout length, and growth inhibition rate under low-temperature stress, the cold-tolerant extreme material px7 and the cold-sensitive extreme material px30 were selected as the core test materials for subsequent experiments. Building on this foundation, the study subjected both extreme materials to artificially simulated low-temperature stress during both the germination and seedling stages, systematically measuring and analyzing the dynamic changes in key physiological and biochemical indicators. This research design not only addresses the limitation of previous studies, which often focused on a single growth stage, but also incorporates low-temperature stress during the harvest period—a factor frequently overlooked in peanut cold tolerance research—into the initial screening criteria. It clarifies the relationship between harvest-period low-temperature stress and cold tolerance during the germination and seedling stages of peanuts, demonstrating distinct research novelty. The ultimate objective of this study is to reveal the physiological response characteristics of cold-tolerant versus cold-sensitive peanut materials to low-temperature stress across different growth stages, identify core indicators for evaluating peanut cold tolerance, and provide essential germplasm resources and theoretical foundations for breeding cold-tolerant peanut varieties.

## 2 Materials and methods

### 2.1 Experimental materials

The 36 peanut materials used in this study were provided by the Peanut Breeding Team of the Liaoning Provincial Institute of Sandland Management and Utilization (details in **Table 1**). All materials were self-bred high-oleic acid peanut varieties.

**Table 1:** 36 Material sources.

Number	Parentage
px1	Jin Hua 26 × Ji Hua 19
px2	Fuhua 12 × HnFxP-4 (selected inbred line)
px3	Faku Sili Hong × Fu Hua 26
px4	Fuhua 22 × Kaibai
px5	Fuhua 12 × ym14 (selected inbred line)
px6	Hua Yu 611 × L11 (line selection)
px7	Fuhua 12 × HnF × P-4 (selected inbred line)
px8	Vn66-6 × Jihua 572
px9	Guihua Hong 95 × Huayu 910
px10	Fuhua 24 × Longhua No. 1
px11	Hua Yu 6805 × HnFxP-2 (line selection)
px12	Huayu 6805 × Kainong 1768
px13	Huayu 9809 × FX6 (Hybrid Selection Line)
px14	Dao 693 × Huayu 662
px15	Jihua 11 × Huayu 917
px16	Fuhua 12th × Jihua 11th
px17	Meilian 2 × CTWE (Hybrid Selection Line)
px18	Tang 8252 × S52 (Hybrid Selection Line)
px19	Jin Hua 26 × Fx6 (Hybrid Selection Line)
px20	Ji Hua 16 × HnFxP-2 (line selection)
px21	Jin Hua 26 × Hua Yu 661
px22	L42E (mutant line selection)
px23	Hua Yu 6805 × HnFxP-2 (Hybrid Selection Line)
px24	Jihua 16 × Huayu 661
px25	Zhen Zhu Hong × S52 (Hybrid Selection Line)
px26	Fu Hua 12 × CTWE (line selection)
px27	Jinhua 19 × Yu Hua 37
px28	Ji Hua 16 × Fx6 (Hybrid Selection Line)
px29	Meilian No. 2 × CTWE (Hybrid Selection Line)
px30	Fuhua 22 × Kaifu 23
px31	Yuan Za 9805 × Hua Yu 32
px32	Jihua 16 × Fx6 (Hybrid Selection Line)
px33	Jinhua 19 × Jihua 19
px34	Hua Yu 6805 × HnFxP-2 (Hybrid Selection Line)
px35	Fuhua No. 4 × CTWE (Hybrid Selection Line)
px36	Fuhua 24 × HnFxP-1 (Hybrid Selection Line)

### 2.2 Experimental methods

#### 2.2.1 Field trials at harvest

The trial was conducted in October 2023 at the Experimental Station of the Liaoning Institute of Sandland Management and Utilization (42°10’N, 122°00’E). Each material was sown over10 m² plots. Soil thermometers (RC-4HC, China Jingchuang) were installed in the field one hour before peanut harvest, recording soil temperature at -5 cm depth hourly. After all materials matured (Sept. 25th), based on weather conditions, peanut plants were pulled from the ground between 9:00–9:30 AM on Oct. 16th. Seed moisture content was measured, and plants were spread flat on the ground for drying. Results are shown in Section 4.1.After cold injury exposure, store field-dried peanuts for 7 days before warehousing. Conduct indoor germination tests in April of the following year. Select 20 uniformly sized seeds per sample, disinfect with 1% NaClO for 10 minutes, then perform germination tests in an intelligent climate chamber (GYJ-150B, GoYoJo, Germany).Seeds were soaked at 26 °C for 12 hours before conducting germination tests on paper. Germination paper was kept moist throughout the process. To assess early seedling development, shoot and root elongation was measured 3 and 7 days post-germination, with each measurement performed in triplicate.

#### 2.2.2 Indoor germination treatment

Building upon the experimental design of previous research (Zhang et al., 2021), this study employed indoor simulated field low-temperature stress tests using peanut materials pre-selected for extreme cold tolerance and extreme cold sensitivity. From each candidate material, 200 plump, uniformly sized seeds were carefully selected and subjected to three distinct low-temperature treatment gradients. The specific stress protocol is as follows. First, peanut seeds were pre-soaked at 26 °C for 12 hours. Subsequently, while ensuring germination paper remained consistently moist, the environmental temperature was lowered at a rate of 2 °C/h to reach target low temperatures of 8 °C, 4 °C, and 0 °C. Upon reaching the target low temperature, seeds were slowly warmed back to 26 °C at a rate of 2 °C/h to resume germination, followed by constant incubation at 26 °C. The total duration of temperature change was 30 hours for the 8 °C treatment group, 34 hours for the 4 °C group, and 38 hours for the 0 °C group. After complete recovery to the normal germination temperature of 26 °C, equal-sized samples were collected at 0, 6, 12, 24, and 36 hours. Collected samples were promptly stored in a -80 °C ultra-low temperature freezer for future use. A normal growth control group (CK) was established concurrently: seeds from the same batch were soaked at 26 °C for 12 hours, then spread on moist germination paper and continuously cultured at a constant 26 °C for 38 hours.

#### 2.2.3 Indoor seedling stage treatment

Selected extreme cold-tolerant and extreme cold-sensitive materials were placed in vermiculite and cultivated in a smart climate chamber under the following conditions: 28 °C/20 °C (day/night), relative humidity of 60%, and a photoperiod of 16h/8h (day/night). Daytime light intensity was maintained at 400 μmol·m^−^²·s^−^¹. After 12 days of cultivation (seedling stage), cold stress treatment was applied. The treatment method followed the protocol in 3.2.2), followed by low-temperature stress treatment after 12 days (seedling stage). Treatment methods refer to Section 3.2.2.

### 2.3 Determination of cold tolerance indicators

Primary indicators measured included: 3-day germination capability 7-day germination capability 3-day shoot length 7-day shoot length Inhibition rate Germination was defined as the emergence of the radicle (first root) from the seed coat. Seedling emergence was defined as the point when the shoot length reached half the root length. Each group consisted of 20 seeds, with the experiment repeated three times.


$$\begin{array}{c} 3-\text{day Germination Capability}=\\ \left(\text{Number of seeds germinated on day }3/\text{Total number of seeds tested}\right)\times 100\% \end{array}$$

$$\begin{array}{c} 7-\text{day Germination capacity}=\\ \left(\text{Number of seeds germinated on day }7/\text{Total number of seeds tested}\right)\times 100\% \end{array}$$

$$\begin{array}{c} 3-\text{day Shoot Length}=\\ \text{Shoot length of seeds germinated on day }3/\text{Number of germinated seeds} \end{array}$$

$$\begin{array}{c} 7\text{d Shoot Length}=\\ \text{Shoot length of seeds germinated on day }7/\text{Number of seeds germinated} \end{array}$$

$$\begin{array}{c} \text{Inhibition rate}=(\text{Sprout length on day }7\text{ under normal cultivation}-\\ \text{Sprout length on day }7\text{ under stress treatment})/\\ \text{Sprout length on day }7\text{ under normal cultivation }*\;\% \end{array}$$

Referencing and optimizing Chen Na’s cold tolerance indicators (Chen and Cheng, 2020), conduct a systematic cold tolerance analysis using germination rate, germination vigor, 3-day shoot length, 7-day shoot length, 3-day shoot-to-root length ratio, and 7-day shoot-to-root length ratio. Assign the best result among each measured indicator as 36 and the worst result as 1. Identify the material with the highest sum of the six measured indicators as cold-tolerant material and the lowest sum as sensitive material.

### 2.4 Enzyme activity assay

Leaf and cotyledon samples were homogenized in a chilled mortar with appropriate extraction buffers, and the supernatants were collected after centrifugation for biochemical assays. All specific assay kits were purchased from Beijing Solarbio Science & Technology Co., Ltd. (Beijing, China), and the procedures briefly followed established classical methods:

SOD Activity: SOD activity was determined using the nitroblue tetrazolium (NBT) photoreduction method (Giannopolitis and Ries, 1977). One unit of SOD activity was defined as the amount of enzyme required to cause 50% inhibition of the NBT photochemical reduction at 560 nm.

POD Activity: POD activity was measured based on the guaiacol oxidation method (Chance and Maehly, 1955). The reaction mixture’s absorbance was monitored at 470 nm, and one unit of enzyme activity was defined as an absorbance change of 0.01 per minute due to the formation of tetraguaiacol.

CAT Activity: CAT activity was assayed by monitoring the consumption of hydrogen peroxide (H_2_O_2_), which correlates with a decrease in absorbance at 240 nm (Aebi, 1984). One unit of CAT activity was defined as the degradation of 1 μmol of H_2_O_2_​ per minute.

MDA, Pro, and Soluble Sugar Contents: MDA content, indicating lipid peroxidation, was quantified using the thiobarbituric acid (TBA) reaction method with absorbance read at 532 nm and 600 nm (Heath and Packer, 1968). Pro content was assessed using the acid-ninhydrin method at 520 nm (Bates et al., 1973). Soluble sugar content was determined via the anthrone-sulfuric acid colorimetric method at 620 nm (Yemm et al., 1954).

### 2.5 Data analysis

The controlled laboratory experiments were arranged in a completely randomized design (CRD) with a factorial treatment structure. All physiological and biochemical measurements were performed using three independent biological replicates. Initial data processing and organization were conducted using Microsoft Excel. Statistical analyses were performed using IBM SPSS Statistics 22.0 (SPSS Inc., Chicago, IL, USA) (IBM Corp, 2013). Data were subjected to a one-way analysis of variance (ANOVA), and significant differences between treatment means were determined using Duncan’s multiple range test at a significance level of P<0.05. Principal component analysis (PCA) and Pearson correlation matrices were executed via GraphPad Prism 10.0 (GraphPad Software, San Diego, CA, USA) (Swift, 1997). All data visualizations and figures were generated using Origin 2025 (OriginLab Corp., Northampton, MA, USA) (OriginLab Corporation, 2025).

## 3 Results

### 3.1 Environmental microclimate analysis during field harvest

**Figure 1:**
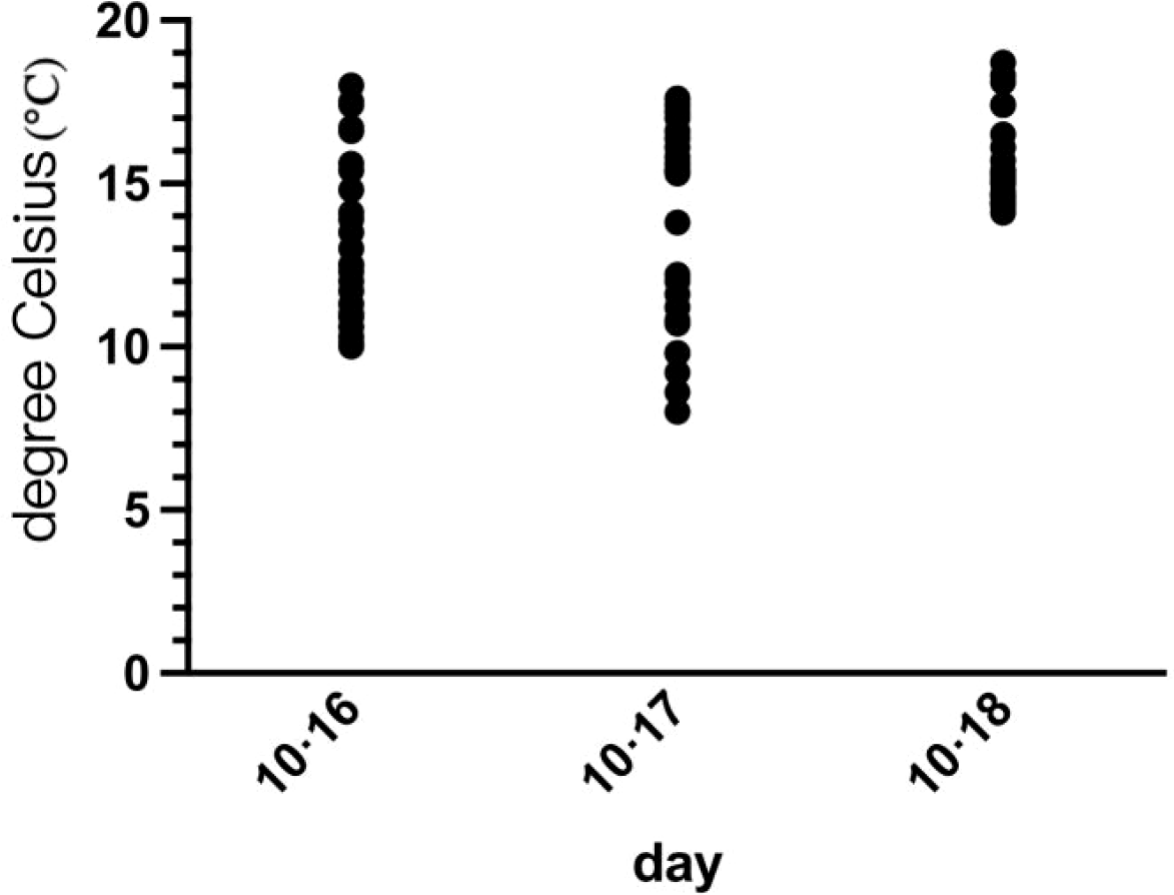
Variations in ground temperature at a depth of -5 cm during the field harvest period from October 16 to October 18, 2023. Data points represent hourly temperature recordings used to determine the low-temperature stress thresholds.

Using a soil thermometer, data were collected from 00:00 on October 16 to 24:00 on October 18. A scatter plot was generated, with results shown in **Figure 1**. Immediately after removal from soil, peanuts exhibited approximately 42% moisture content. As shown in **Figure 1**, the lowest temperature recorded at 5 cm below soil surface on the evening of October 17 reached 8 °C. At this point, peanuts were subjected to low-temperature stress. Consequently, we conducted field low-temperature stress treatments on 36 samples from October 16 to October 18.

### 3.2 Identification and pedigree analysis of extreme cold-tolerant genotypes

After 36 peanut accessions were field-dried and subjected to natural low-temperature stress for 3 days, significant genotypic differences in germination were observed (**Table 2**). Overall, the 3-day sprout length ranged from 0 to 2.66 cm, while the 7-day sprout length ranged from 0 to 5.43 cm; while the sprout length ratios at 3d and 7d ranged from 0 to 1.44 and 0 to 3.32, respectively. To accurately assess cold tolerance potential, this study focused on analyzing the inhibition rate of cold treatment on sprout length at day 7. Results indicated that material px7 exhibited the lowest inhibition level, demonstrating exceptional cold tolerance; while px30 and px3 exhibited the highest inhibition rates, indicating extreme sensitivity to low temperatures. Considering that under normal optimal cultivation conditions, px30’s baseline bud length significantly outperformed px3, selecting the inherently vigorous px30 as the material more accurately reflects growth arrest directly caused by cold damage, eliminating interference from inherent weak vitality in the material. Therefore, this study ultimately established px7 as the candidate material for extreme cold tolerance and px30 as the candidate material for extreme cold sensitivity for subsequent in-depth physiological and biochemical testing.

**Table 2:** Screening data of 36 materials.

Variety	A 3-day germination capacity	A 7-day germination capacity	B 3-day germination capacity	B 7-day germination capacity	A 3-day sprout length	A 7-day sprout length	B 3-day sprout length	B 7-day sprout length	Inhibition rate
px1	50%	70%	70%	85%	0.29 ± 0.2	2.54 ± 0.51	0.55 ± 0.38	3.35 ± 0.67	24%
px2	35%	55%	45%	55%	0.74 ± 0.13	2.31 ± 0.48	1.40 ± 0.25	3.03 ± 0.63	24%
px3	0%	0%	50%	60%	0 ± 0	0 ± 0	0.92 ± 0.15	2.78 ± 0.12	100%
px4	10%	10%	40%	40%	0.28 ± 0.19	1.16 ± 0.39	0.53 ± 0.36	1.52 ± 0.51	24%
px5	40%	50%	55%	60%	0.75 ± 0.13	1.69 ± 0.47	1.42 ± 0.25	2.27 ± 0.63	26%
px6	20%	20%	45%	55%	0.59 ± 0.13	1.42 ± 0.40	1.11 ± 0.25	1.89 ± 0.53	25%
px7	100%	100%	95%	100%	2.26 ± 0.37	5.39 ± 0.71	4.26 ± 0.70	6.02 ± 0.92	10%
px8	5%	5%	45%	65%	0.38 ± 0.2	0.70 ± 0.40	0.72 ± 0.38	0.95 ± 0.54	26%
px9	35%	55%	45%	55%	0.77 ± 0.14	0.95 ± 0.40	1.45 ± 0.26	1.26 ± 0.53	25%
px10	55%	70%	60%	75%	2.41 ± 0.22	4.99 ± 0.71	4.55 ± 0.42	6.66 ± 0.95	25%
px11	75%	80%	75%	75%	0.77 ± 0.14	2.46 ± 0.49	1.45 ± 0.26	3.23 ± 0.64	24%
px12	65%	65%	70%	75%	1 ± 0.26	1.03 ± 0.41	1.89 ± 0.49	1.38 ± 0.55	25%
px13	35%	75%	45%	65%	1.27 ± 0.16	4.39 ± 0.70	2.39 ± 0.30	5.78 ± 0.92	24%
px14	15%	20%	50%	55%	0.51 ± 0.21	1.10 ± 0.41	0.96 ± 0.40	1.44 ± 0.54	24%
px15	40%	40%	50%	50%	2.43 ± 0.22	3.14 ± 0.57	4.58 ± 0.42	4.23 ± 0.77	26%
px16	20%	20%	35%	55%	0.79 ± 0.24	1.16 ± 0.41	1.49 ± 0.45	1.53 ± 0.54	24%
px17	70%	75%	65%	65%	1.84 ± 0.33	4.61 ± 0.73	3.47 ± 0.62	6.06 ± 0.96	24%
px18	45%	50%	50%	75%	1.29 ± 0.28	3.06 ± 0.57	2.43 ± 0.53	4.10 ± 0.76	25%
px19	60%	75%	75%	80%	1.84 ± 0.19	5.46 ± 0.73	3.47 ± 0.36	7.24 ± 0.97	25%
px20	30%	30%	50%	65%	1.34 ± 0.29	3.11 ± 0.57	2.53 ± 0.55	4.07 ± 0.75	24%
px21	10%	25%	55%	60%	0.44 ± 0.21	1.32 ± 0.41	0.83 ± 0.40	1.78 ± 0.55	26%
px22	0%	20%	40%	45%	0 ± 0	1.27 ± 0.40	0.89 ± 0.14	1.68 ± 0.53	24%
px23	45%	60%	55%	60%	1.22 ± 0.16	3.62 ± 0.62	2.30 ± 0.30	4.75 ± 0.81	24%
px24	15%	30%	45%	55%	1.74 ± 0.32	2.05 ± 0.48	3.28 ± 0.60	2.74 ± 0.64	25%
px25	60%	65%	60%	75%	1.49 ± 0.17	3.55 ± 0.62	2.81 ± 0.32	4.68 ± 0.82	24%
px26	25%	80%	70%	75%	0.77 ± 0.14	4.50 ± 0.71	1.45 ± 0.26	5.90 ± 0.93	24%
px27	60%	75%	70%	85%	2.66 ± 0.4	4.55 ± 0.71	5.02 ± 0.75	6.12 ± 0.95	26%
px28	70%	75%	85%	100%	1.88 ± 0.19	3.72 ± 0.59	3.55 ± 0.36	4.95 ± 0.79	25%
px29	10%	20%	45%	60%	0.73 ± 0.13	1.57 ± 0.41	1.38 ± 0.25	2.06 ± 0.54	24%
px30	0%	0%	85%	90%	0 ± 0	0 ± 0	1.95 ± 0.16	5.85 ± 0.17	100%
px31	25%	50%	55%	70%	0.78 ± 0.14	1.45 ± 0.41	1.47 ± 0.26	1.95 ± 0.55	26%
px32	30%	30%	60%	65%	0.61 ± 0.22	1.40 ± 0.40	1.15 ± 0.42	1.84 ± 0.53	24%
px33	20%	25%	50%	65%	0.34 ± 0.12	1.77 ± 0.48	0.64 ± 0.23	2.34 ± 0.64	24%
px34	15%	15%	50%	55%	0.82 ± 0.14	1.46 ± 0.41	1.55 ± 0.26	1.95 ± 0.55	25%
px35	5%	5%	45%	55%	0.57 ± 0.13	0.58 ± 0.40	1.08 ± 0.25	0.76 ± 0.52	24%
px36	10%	15%	55%	75%	1.25 ± 0.16	2.67 ± 0.56	2.36 ± 0.30	3.56 ± 0.75	25%

A represents peanut germination experiments conducted under low-temperature stress conditions, while B denotes the standard deviation of peanut germination experiments conducted under non-stress conditions. The data in the table reflects the standard deviation of 20 seeds from three replicate experiments.

The extreme phenotypic contrast exhibited by these two materials under low temperatures has a clear genetic basis. Tracing their pedigree reveals that the cold-tolerant material PX7 has the parental combination ‘Fuhua 12 × HnFxP-4 (selected line)’, while the cold-sensitive material PX30 has the parental combination ‘Fuhua 22 × Kaifu 23’. Their fundamentally distinct parental origins conferred highly differentiated genotypes. This inherent genetic divergence formed the basis for their significant physiological and biochemical differentiation in subsequent low-temperature stress responses, specifically in the activation rate of antioxidant enzyme systems and the accumulation capacity of osmotic regulatory substances.

### 3.3 Dynamics of membrane lipid peroxidation and osmotic adjustment under cold stress

**Figure 2:**
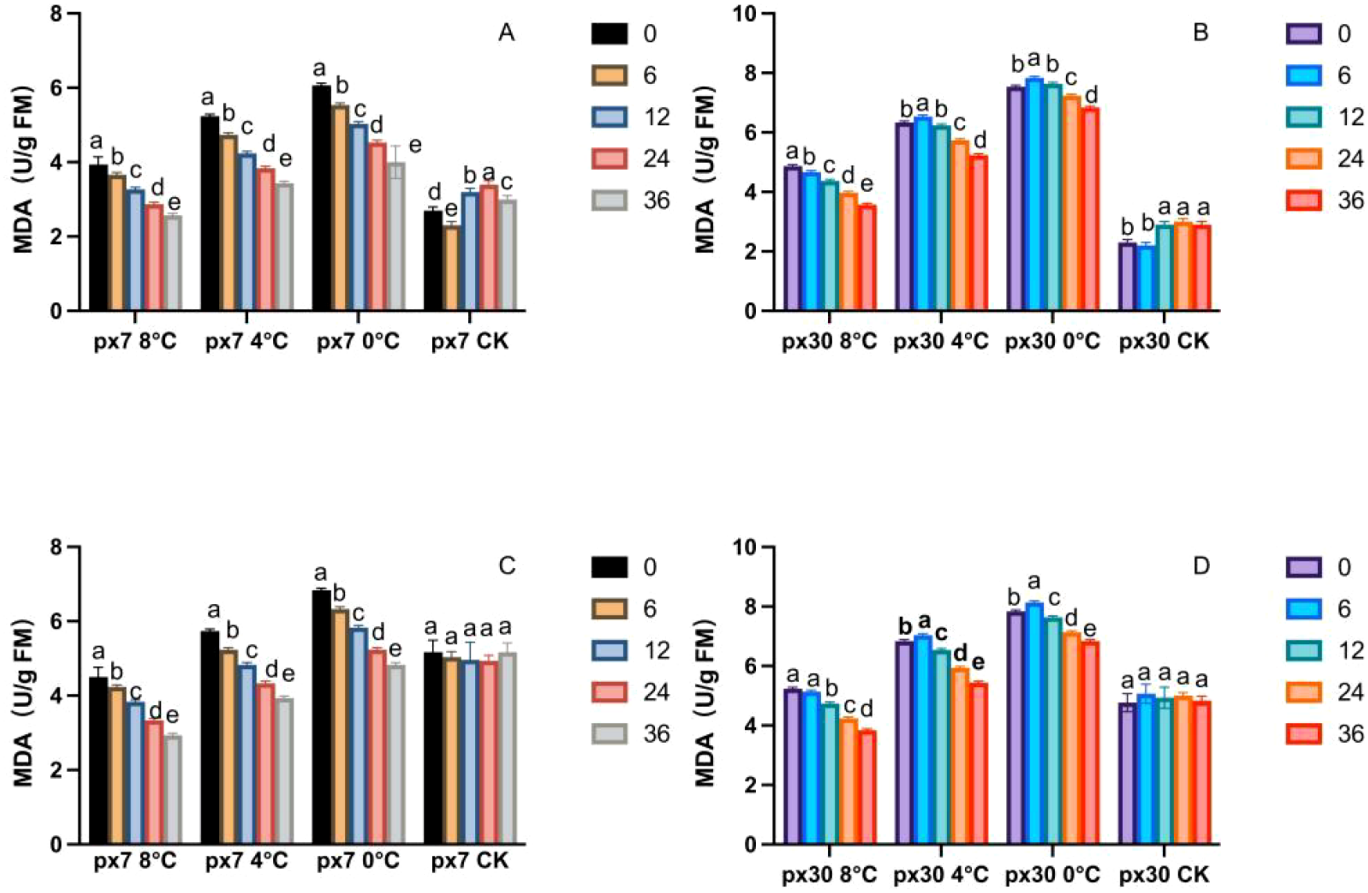
Trends in MDA content variation over 36 hours of recovery post-cold stress. **(A)** Cotyledons of the cold-resistant variety during germination; **(B)** cotyledons of the cold-sensitive variety during germination; **(C)** leaves of the cold-resistant seedlings; **(D)** leaves of the cold-sensitive seedlings. Letters within the same group denote significant differences between treatment durations (P<0.05) according to Duncan’s multiple range test.

As shown in **Figure 2**, the horizontal axis px7 8°C denotes cold-resistant material (px7) treated to achieve a minimum low-temperature stress threshold of 8°C; px7 4°C denotes cold-resistant material (px7) treated to achieve a minimum low-temperature stress threshold of 4°C; px7 0°C denotes cold-resistant material (px7) treated to achieve a minimum low-temperature stress threshold of 0°C; px7 CK denotes the control group. (px30 denotes sensitive material, following the same principle) The vertical axis represents MDA content (same applies to Pro, soluble sugars, CAT, POD, and SOD below). Letters within the same group indicate significant differences.

Following the conclusion of the indoor simulated low-temperature stress treatment, the significance analysis in **Figure 2** indicates that MDA content exhibited a significant overall downward trend (P< 0.05) for both sensitive and cold-tolerant varieties, regardless of whether they were in the peanut germination stage or seedling stage. Excluding the control group, MDA levels peaked at 0h or 6h (a) and significantly decreased to their lowest point at 36h (P< 0.05). Moreover, during the germination stage, MDA content in sensitive materials was significantly higher than in cold-tolerant materials (P< 0.05). At 8 °C, sensitive materials exhibited MDA levels 20%–35% higher than cold-tolerant materials from 0 to 36 hours; at 4 °C, this difference increased to 23%–51%; and at 0 °C, it rose to 27%–64%. During the seedling stage, under 8 °C treatment, MDA content in sensitive materials was 13%–34% higher than in cold-tolerant materials from 0 to 36 hours; under 4 °C treatment, it was 21%–38% higher; and under 0 °C treatment, it was 14%–41% higher. (Detailed experimental results are provided in the appendix.)

**Figure 3:**
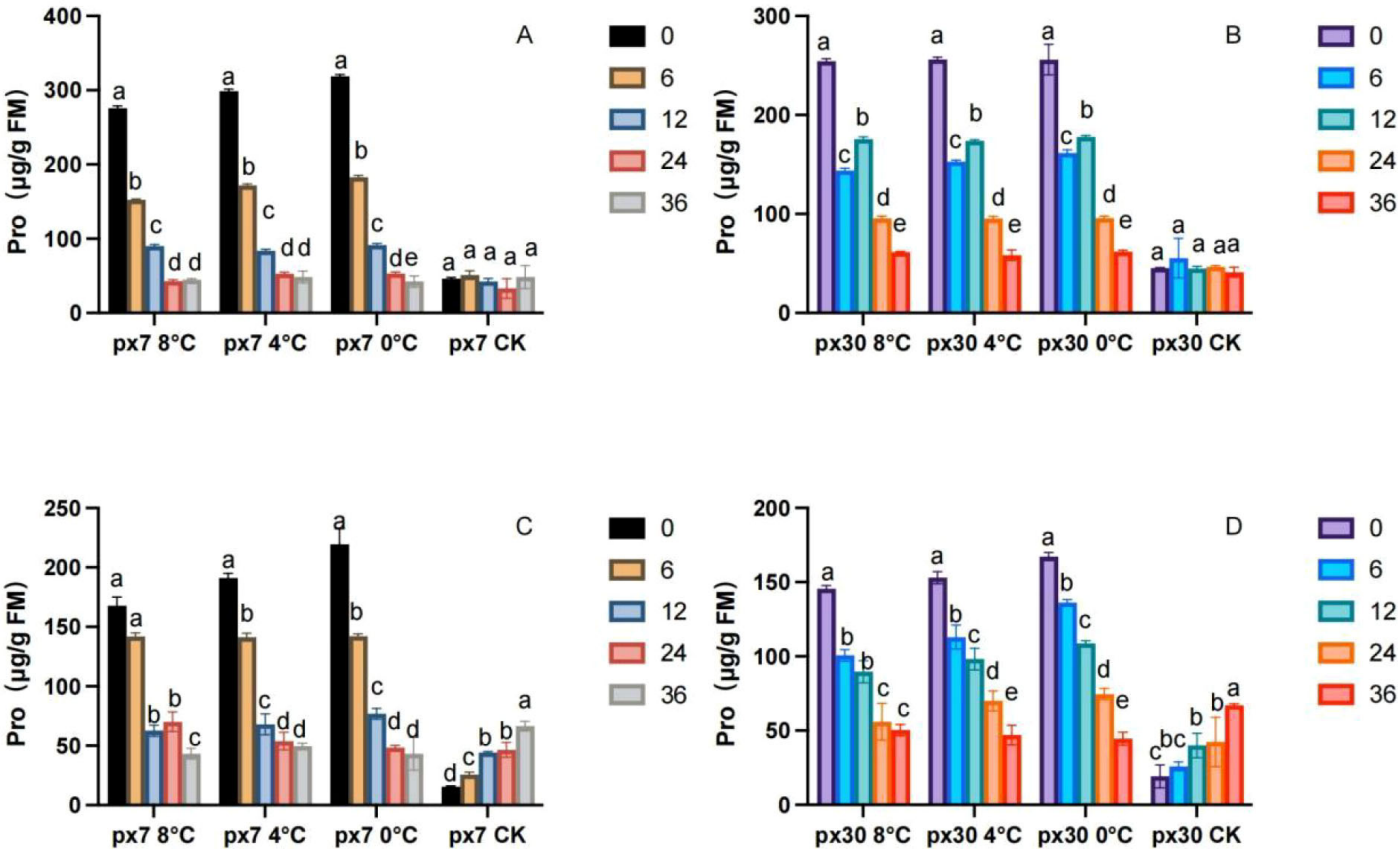
Trends in Pro content variation over 36 hours of recovery post-cold stress. **(A)** Cotyledons of the cold-resistant variety during germination; **(B)** cotyledons of the cold-sensitive variety during germination; **(C)** leaves of the cold-resistant seedlings; **(D)** leaves of the cold-sensitive seedlings. Letters within the same group denote significant differences between treatment durations (P<0.05) according to Duncan’s multiple range test.

As shown in **Figure 3**, following the conclusion of indoor simulated low-temperature stress on peanuts, Pro content exhibited a significant downward trend during both the germination and seedling stages (P< 0.05). After the stress period ended, Pro content peaked at 0 hours across all treatments and developmental stages for both materials (a), followed by a sustained significant decline (P< 0.05), reaching its lowest point at 36 hours. Overall, the sensitive material exhibited significantly lower Pro content than the cold-tolerant material (P< 0.05). During the germination stage, the sensitive material showed Pro levels 9.5% to 0.0% lower than the cold-tolerant material at 8 °C from 0 to 36 hours, 14.3% to 12.3% lower at 4 °C, and 17.9% to 16.6% lower at 0 °C. During the seedling stage, Pro content in sensitive materials was 16.3%–22.3% lower than in cold-tolerant materials at 8 °C for 0–36 hours; 21.3%–16.4% lower at 4 °C; and 27.4%–12.6% lower at 0 °C.

**Figure 4:**
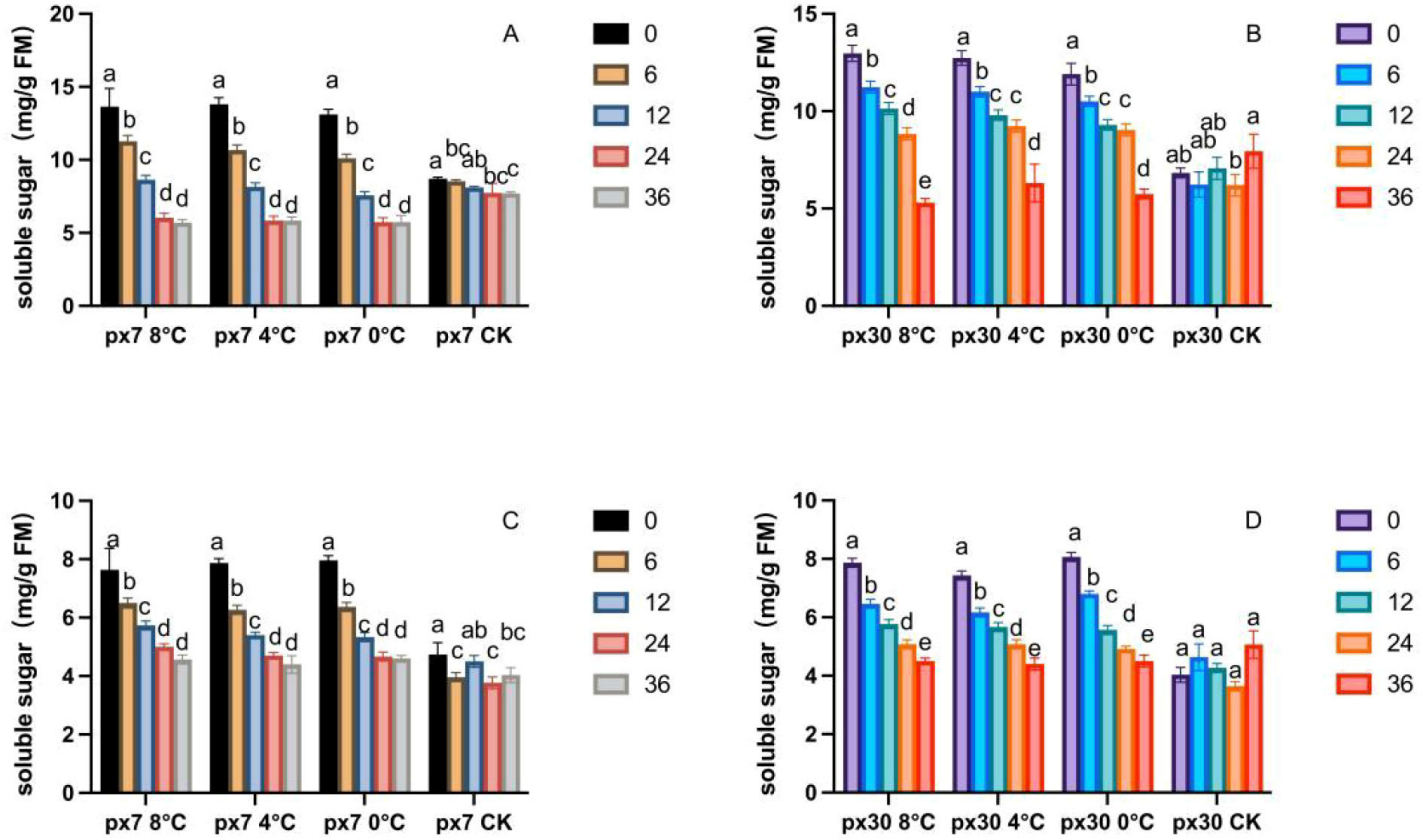
Trends in soluble sugar content variation over 36 hours of recovery post-cold stress. **(A)** Cotyledons of the cold-resistant variety during germination; **(B)** cotyledons of the cold-sensitive variety during germination; **(C)** leaves of the cold-resistant seedlings; **(D)** leaves of the cold-sensitive seedlings. Letters within the same group denote significant differences between treatment durations (P<0.05) according to Duncan’s multiple range test.

As shown in **Figure 4**, following the conclusion of the simulated low-temperature stress treatment indoors, the soluble sugar content in peanuts exhibited a sustained and significant downward trend (P< 0.05) during both the germination and seedling stages. Significance analysis revealed that for both materials, soluble sugar content reached its peak at 0 hours (a) after the end of stress under different low-temperature treatments and developmental stages, and then gradually decreased significantly (P< 0.05) during recovery, reaching the lowest level at 36 hours. During the germination stage, the sensitive material exhibited significantly lower soluble sugar content than the cold-tolerant material under 8 °C treatment (P< 0.05), with reductions ranging from 33.27% to 40.25% between 0 and 36 hours. Under 4 °C treatment, the sensitive material showed reductions of 30.08% to 38.15%. and at 0 °C, they were 31.11%–41.87% lower. During the seedling stage at 8 °C treatment, soluble sugar content in sensitive materials was 1.27%–9.48% lower than in cold-tolerant materials at 0–36 hours; at 4 °C treatment, sensitive materials showed 12.02%–20.34% lower levels; at 0 °C treatment, sensitive materials exhibited 19.04%–29.48% lower levels.

### 3.4 Synergistic variations in the antioxidant enzyme system during recovery phase

**Figure 5:**
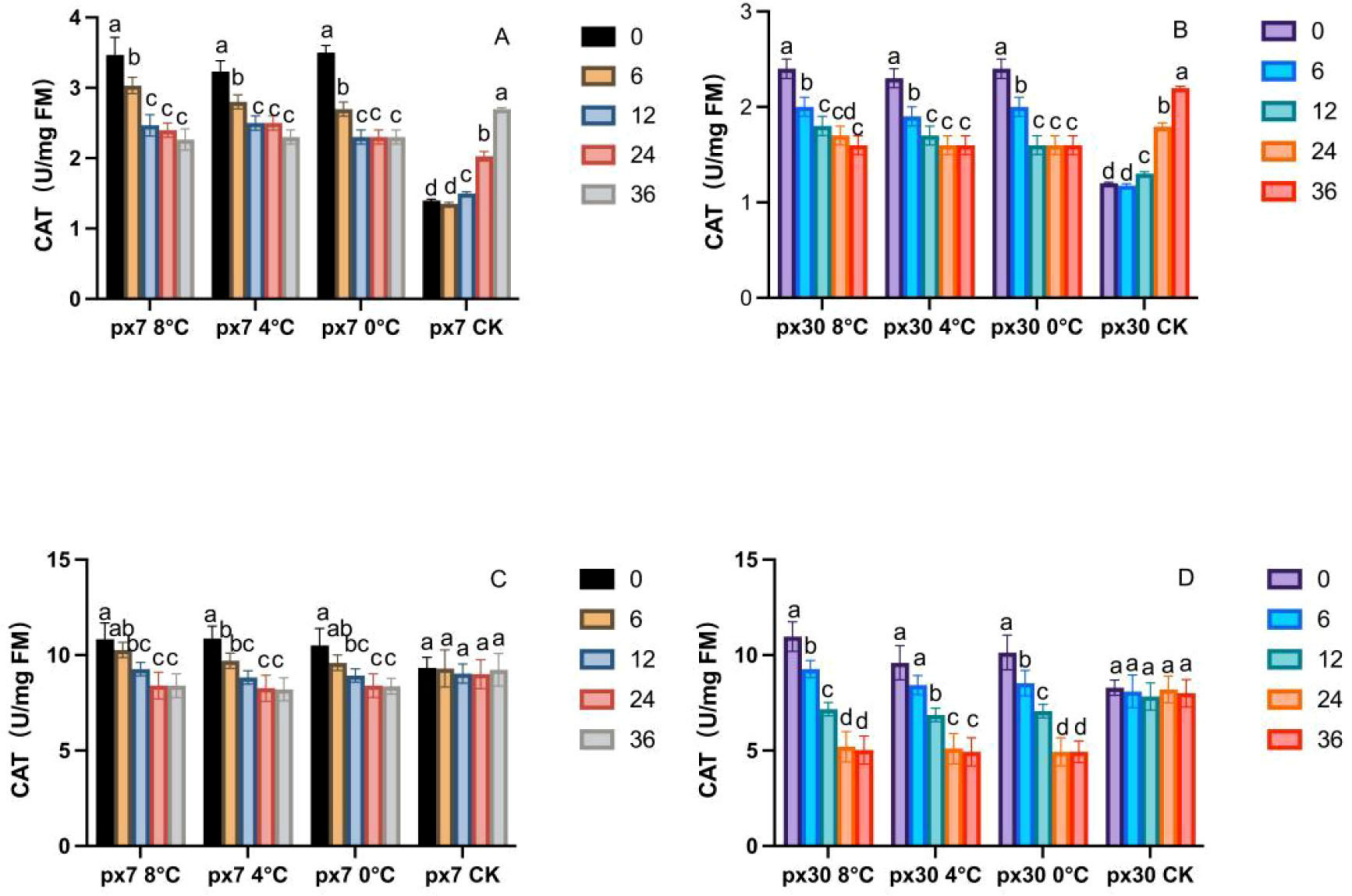
Trends in CAT content variation over 36 hours of recovery post-cold stress. **(A)** Cotyledons of the cold-resistant variety during germination; **(B)** cotyledons of the cold-sensitive variety during germination; **(C)** leaves of the cold-resistant seedlings; **(D)** leaves of the cold-sensitive seedlings. Letters within the same group denote significant differences between treatment durations (P<0.05) according to Duncan’s multiple range test.

**Figure 5** shows that following the conclusion of simulated low-temperature stress indoors, CAT activity exhibited a sustained and significant decline (P< 0.05) during both the germination and seedling stages of peanuts. For both materials, CAT activity peaked at 0 hours post-stress termination (a) under different low-temperature treatments and developmental stages, then decreased significantly to its lowest level by 36 hours (P< 0.05). During the germination stage, CAT activity in the sensitive material was significantly lower than that in the cold-tolerant material under the 8 °C treatment (P< 0.05), showing a reduction of 33.27%–40.25% from 0 to 36 hours. Under the 4 °C treatment, the sensitive material exhibited a 30.08%–38.15% decrease, while under the 0 °C treatment, it showed a reduction of 31.11%–41.87%. During the seedling stage, CAT activity in sensitive materials was 1.27%–9.48% lower than in cold-tolerant materials at 8 °C for 0–36 hours. At 4 °C, sensitive materials showed 12.02%–20.34% lower activity, while at 0 °C, activity decreased by 19.04%–29.48%.

**Figure 6:**
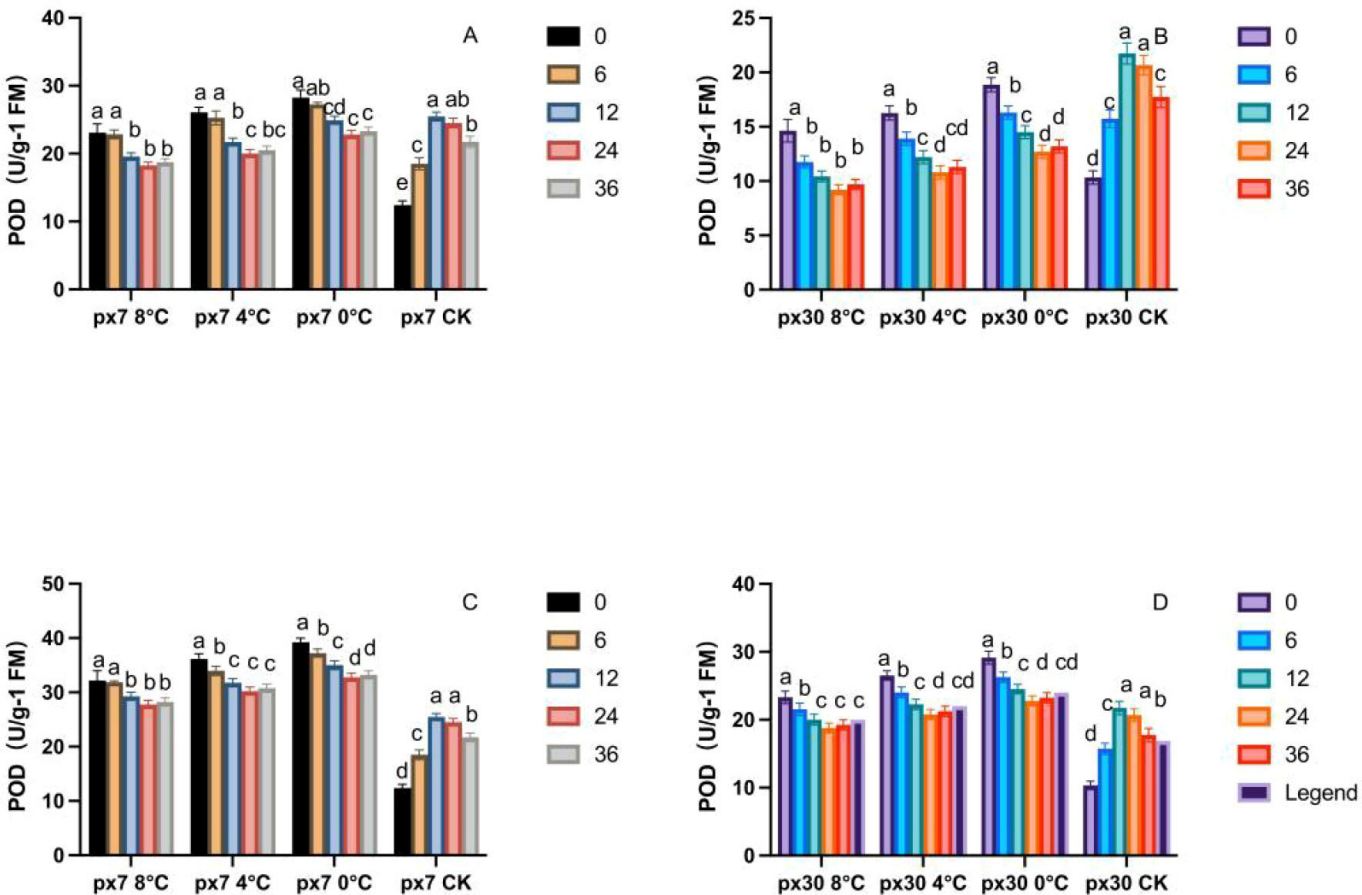
Trends in POD content variation over 36 hours of recovery post-cold stress. **(A)** Cotyledons of the cold-resistant variety during germination; **(B)** cotyledons of the cold-sensitive variety during germination; **(C)** leaves of the cold-resistant seedlings; **(D)** leaves of the cold-sensitive seedlings. Letters within the same group denote significant differences between treatment durations (P<0.05) according to Duncan’s multiple range test.

As shown in **Figure 6**, following the conclusion of simulated low-temperature stress indoors, both peanut germination-stage and seedling-stage POD activity exhibited a sustained, significant downward trend (P< 0.05). For both materials, under different low-temperature treatments and developmental stages, POD activity peaked at 0 hours post-stress termination (a) and significantly declined to its lowest level by 36 hours (P< 0.05). During the germination stage under 8 °C treatment, POD activity in the sensitive material exhibited significant differences (P< 0.05) compared to the cold-tolerant material, with differences ranging from -26.8% to 46.6% between 0 and 36 hours. Under 4 °C treatment, the sensitive material exhibited significantly lower POD activity (P< 0.05) by 38.3%–43.3%, while under 0 °C treatment, it was 36.1%–40.9% lower. During the seedling stage, at 8 °C treatment, POD activity in sensitive materials was significantly lower than in cold-tolerant materials at 0–36 h (P< 0.05), ranging from 31.4% to 30.8%. At 4 °C treatment, sensitive materials showed lower activity by 26.5% to 29.4%; at 0 °C treatment, sensitive materials were lower by 27.0% to 29.3%.

**Figure 7:**
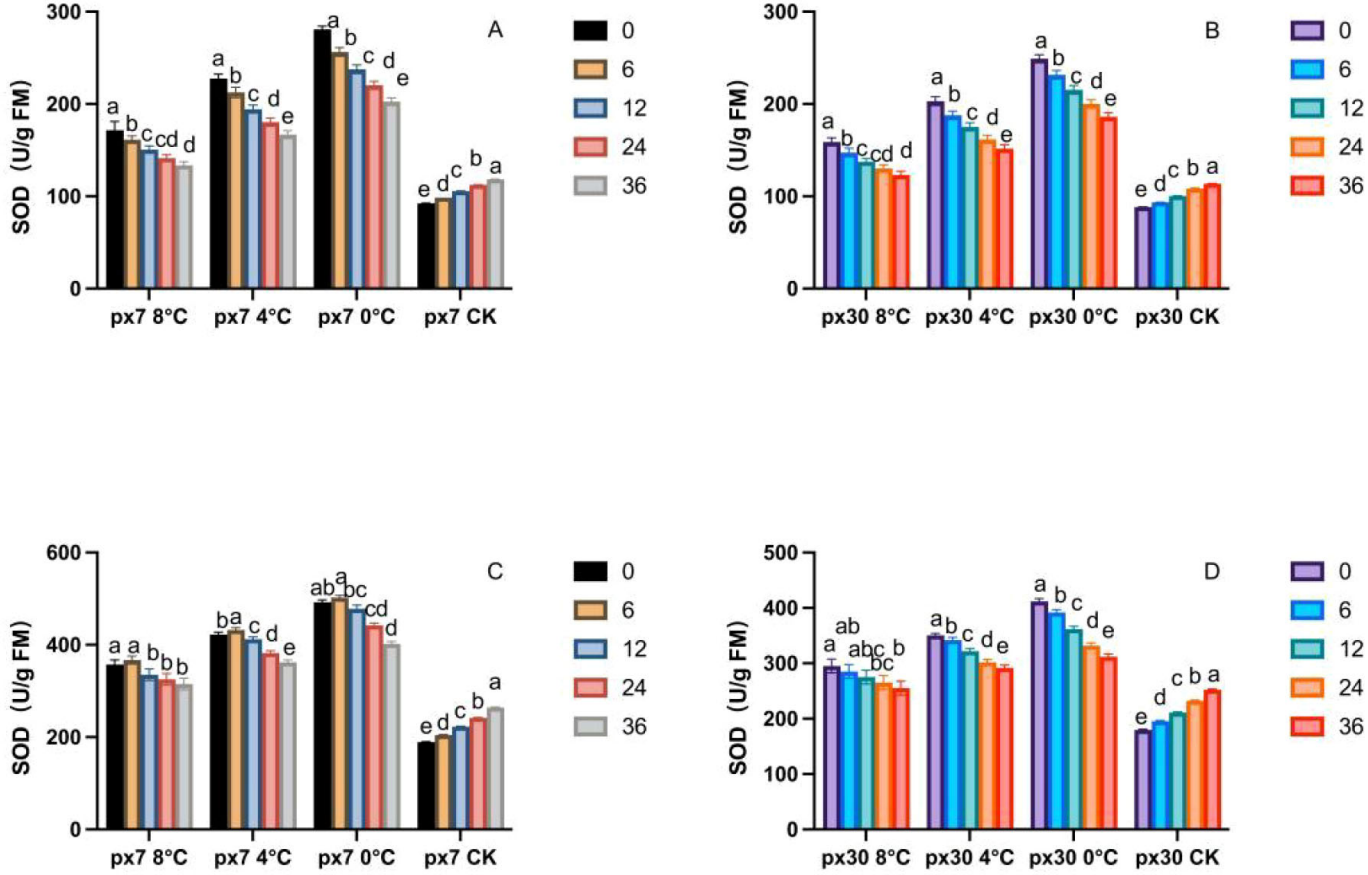
Trends in SOD content variation over 36 hours of recovery post-cold stress. **(A)** Cotyledons of the cold-resistant variety during germination; **(B)** cotyledons of the cold-sensitive variety during germination; **(C)** leaves of the cold-resistant seedlings; **(D)** leaves of the cold-sensitive seedlings. Letters within the same group denote significant differences between treatment durations (P<0.05) according to Duncan’s multiple range test.

As shown in **Figure 7**, following the conclusion of simulated low-temperature stress indoors, both peanut germination and seedling stages exhibited a sustained, significant decline in SOD activity (P< 0.05). SOD activity peaked at 0 hours post-stress termination (a) and significantly decreased to its lowest level by 36 hours (P< 0.05). Overall, SOD activity in sensitive materials was significantly lower than in cold-tolerant materials (P< 0.05). During the germination stage, under 8 °C treatment, the SOD activity of sensitive materials was 7.9%–9.3% lower than that of cold-tolerant materials from 0 to 36 hours. Under 4 °C treatment, sensitive materials showed a 9.1%–11.6% reduction, and under 0 °C treatment, a 8.1%–11.3% decrease. During the seedling stage, SOD activity in sensitive materials was 16.9%–20.4% lower than in cold-tolerant materials at 8 °C for 0–36 hours; at 4 °C, it was 16.9%–21.8% lower; and at 0 °C, it was 16.3%–24.9% lower.

### 3.5 Integrated assessment and PCA-based evaluation of cold-tolerance indicators

**Figure 8:**
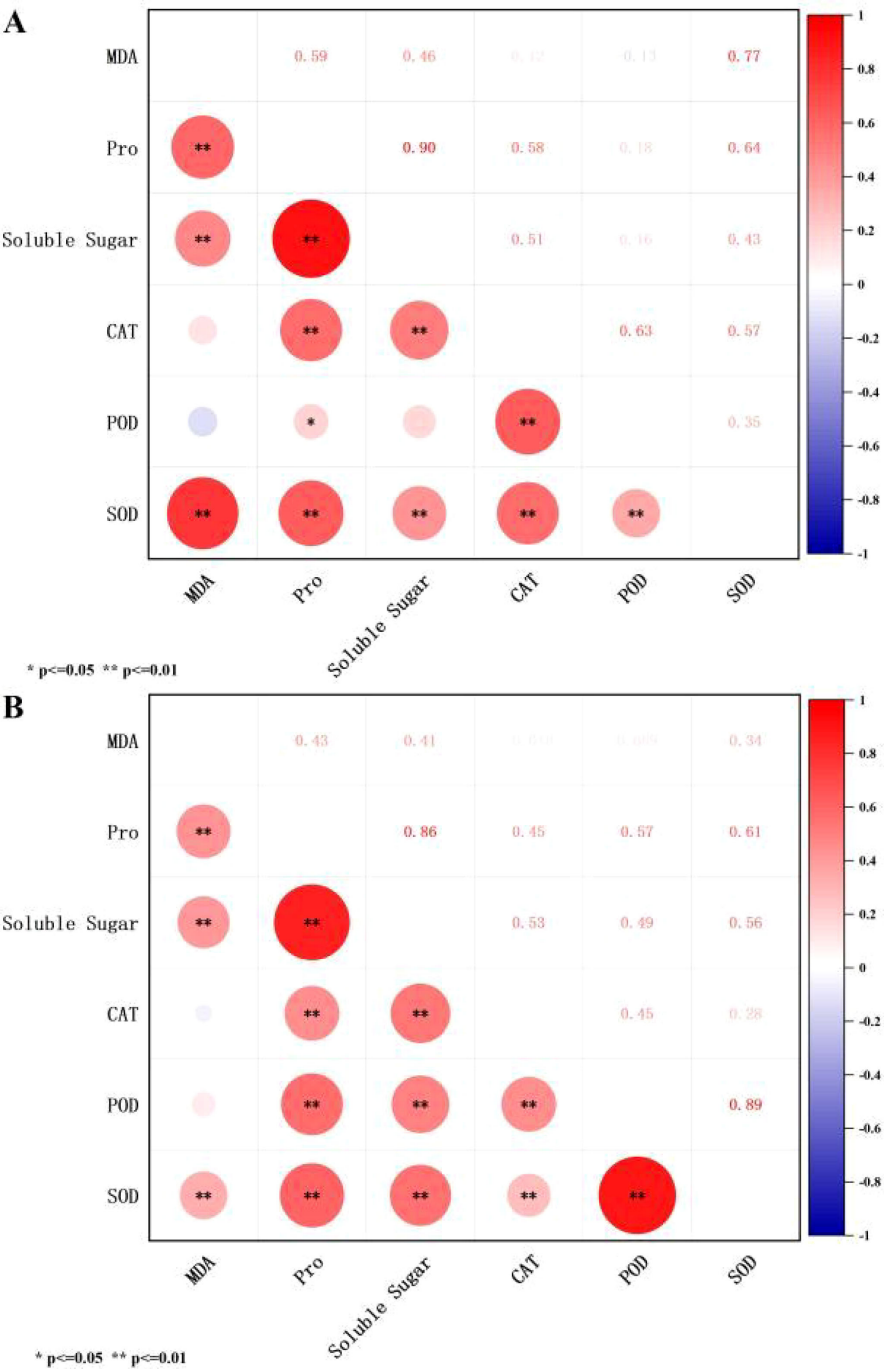
Correlation test of cold resistance indicators Note: **(A)** denotes the germination stage of peanuts; **(B)** denotes the seedling stage of peanuts.

As can be seen in **Figure 8**, Further analysis of correlations among indicators during peanut germination and seedling stages revealed significant positive correlations between Pro, soluble sugars, SOD, and MDA in both stages. All indicators showed significant or extremely significant positive correlations with Pro. CAT and SOD exhibited extremely significant positive correlations with soluble sugars, while POD and SOD showed extremely significant correlations with CTA. SOD and POD also demonstrated extremely significant correlations.Notably, POD showed a highly significant positive correlation with soluble sugars during the seedling stage, whereas no significant positive correlation existed between POD and soluble sugars during germination. Correlation coefficients indicated strong associations and information overlap among traits, highlighting limitations of single-indicator evaluation. Thus, dimensionality reduction via principal component analysis was employed for comprehensive assessment to mitigate measurement errors.

To quantitatively evaluate six multidimensional physiological traits of extremely cold-tolerant (px7) and cold-sensitive (px30) peanut materials at two developmental stages, PCA was conducted in this study. Based on the mathematical criterion of eigenvalues > 1, three principal components (PCs) were extracted, contributing 56.12%, 20.20%, and 13.02% of the variance, respectively, with a cumulative variance contribution of 89.35%. This indicates that the first three principal components effectively captured and represented the majority of information in the original data matrix.

**Figure 9:**
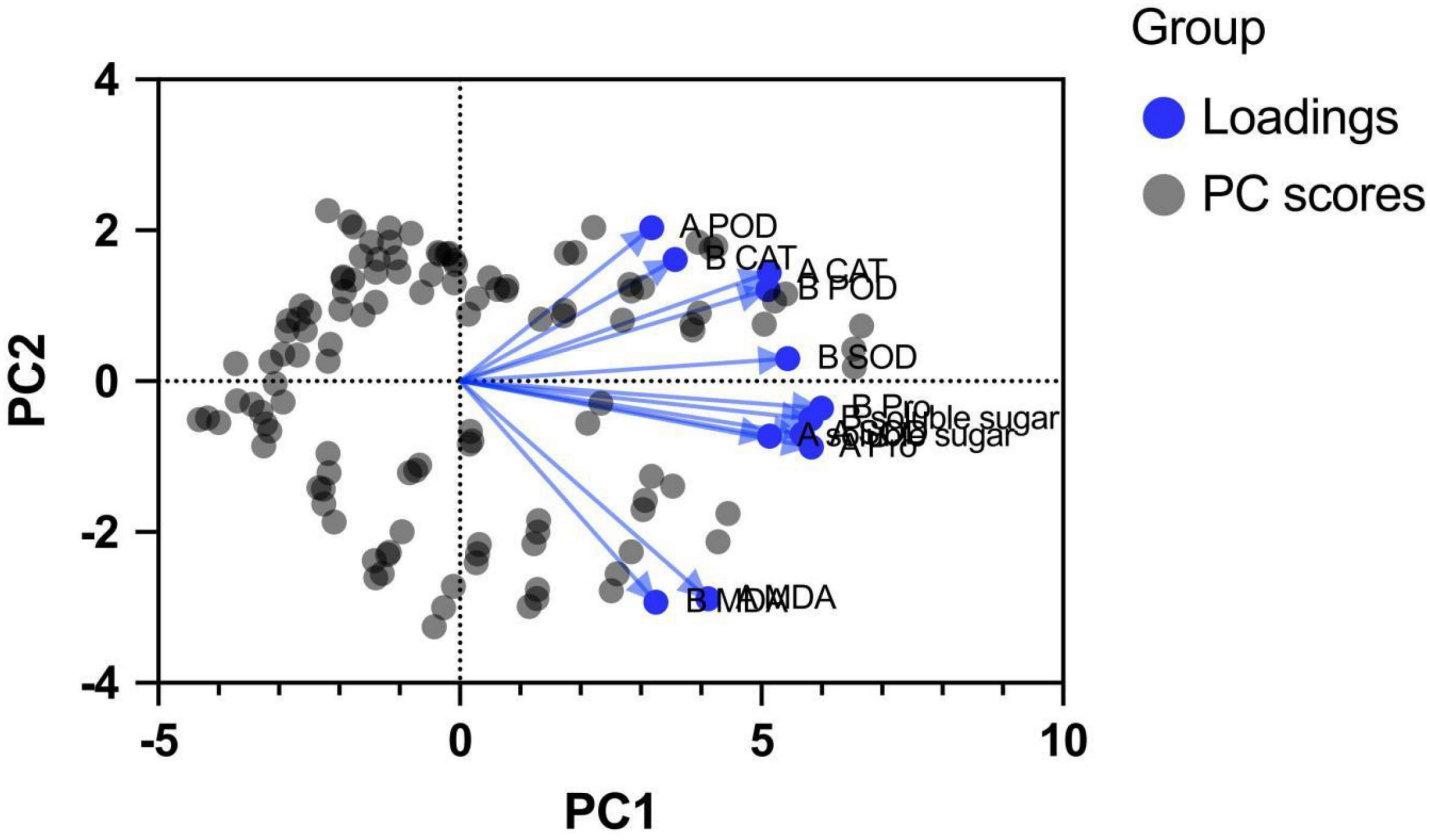
PCA loading plot of physiological indicators in peanuts. Note: The vectors represent the loading scores of each indicator on PC1 and PC2. Indicators clustered toward the right (Pro, SOD, Sugars) are the primary factors contributing to cold tolerance differentiation.

As shown in **Figure 9**, the distribution of physiological indicators in the first principal component (PC1) and second principal component (PC2) spaces exhibited distinct clustering patterns. According to the factor loading matrix (**Table 3**), PC1 was primarily driven by Pro, SOD, and soluble sugar content. Specifically, the loadings of Pro at the seedling stage, Pro at the germination stage, soluble sugars at the seedling stage, and SOD at both the germination and seedling stages on PC1 reached 0.906, 0.881, 0.880, 0.858, and 0.822, respectively. In **Figure 9**, the vectors corresponding to these indicators are tightly clustered along the positive direction of the PC1 axis and exhibit the longest lengths, visually demonstrating their role as core evaluation indicators for distinguishing materials with different cold tolerance potentials.

**Table 3:** Principal component analysis of cold resistance index.

Index	PC1	PC2	PC3
A MDA	0.622	-0.706	0.275
B MDA	0.491	-0.718	0.133
A Pro	0.881	-0.214	-0.351
B Pro	0.906	-0.088	-0.221
A soluble sugar	0.776	-0.179	-0.555
B soluble sugar	0.88	-0.123	-0.347
A CAT	0.776	0.505	0.003
B CAT	0.54	0.566	-0.422
A POD	0.481	0.715	0.188
B POD	0.772	0.426	0.426
A SOD	0.858	-0.173	0.459
B SOD	0.822	0.104	0.514
Eigenvalue	6.735	2.424	1.563
Cumulative (%)	56.12	20.2	13.02
Cumulative proportion of variance (%)	56.12	76.32	89.35

A represents the germination stage of peanuts; B represents the seedling stage of peanuts.

In contrast, MDA exhibited significant negative loadings on PC2 (-0.706 during germination and -0.718 during seedling stages), with its vector pointing downward in the loading plot—forming a stark contrast to the vector directions of CAT and POD (both positive on PC2). This spatial distribution pattern reveals a physiological antagonistic relationship between membrane lipid peroxidation levels and the antioxidant defense system.

To precisely quantify the cold tolerance of two extreme peanut genotypes, this study constructed a comprehensive evaluation mathematical model based on PCA variance contribution rates: F = (56.12% × PC1 + 20.20% × PC2 + 13.02% × PC3)/89.35%. Calculations show that the comprehensive evaluation score of the highly cold-tolerant material px7 (F = 1.63) is significantly higher than that of the cold-sensitive material px30 (F=−1.36). This quantitative assessment based on multidimensional physiological and biochemical data strongly correlates with phenotypic identification results from field natural low-temperature stress and indoor germination tests, mathematically demonstrating that px7 possesses a superior physiological resistance foundation under low-temperature stress.

## 4 Discussion

Peanuts grown in high-latitude regions are prone to quality degradation if harvested late or encountered by early frost, which impairs seed germination the following year and complicates seed retention (Zhang and Dong, 2019). Screening cold-tolerant germplasm represents the most effective strategy to address low-temperature stress challenges in peanuts (de Freitas and Thomas, 2019; Zhang and Jiang, 2021; Su and Tan, 2022; Shikha and Thayil, 2023). Regarding low-temperature stress selection, this study monitored soil temperatures during harvest, which dropped as low as 8 °C. Combined with daily weather forecasts (https://weather.cma.cn/web/weather/), it was determined that nighttime lows frequently reached 1 °C during harvest. Soil thermometer measurements confirmed that in previous years, peanut kernels buried deep underground often suffered cold damage below 10 °C during harvest. Furthermore, China Meteorological Network indicated that the lowest temperature encountered by the 36 field trial materials in the field was 1 °C. Therefore, this laboratory set simulated low temperatures of 8 °C, 4 °C, and 0 °C as low-temperature stress conditions, with a temperature gradient of 2 °C/h, which more closely approximates field temperature changes compared to previous studies (Tang and Wang, 2011). During the evaluation of cold-tolerant peanut materials, this study observed that freshly harvested peanuts subjected to light frost stress exhibited no initial inhibition of seed germination but experienced significant subsequent growth retardation. Therefore, this study focused on analyzing low-temperature responses during early germination rather than strictly adhering to the International Seed Testing Association (ISTA) standards. By using differences between stress and normal cultivation conditions as screening criteria, it effectively distinguished the cold-tolerant material px7 from the sensitive material px30. Subsequent physiological indicator results further supported the validity of this cold-tolerance screening approach.

Cold stress induces degradation of cell membrane fluidity, leading to lipid peroxidation (Hasanuzzaman and Bhuyan, 2020; Panda and Gupta, 2024). Han et al. observed varying degrees of MDA increase in peanut seedlings after rice seedlings experienced low temperatures (Han and Huang, 2017). Our study indicates that peanut cotyledons during germination exhibit similar patterns to leaves. For MDA content in seedlings, the sensitive material showed changes ranging from 8% to -20% under 8 °C treatment; under 4 °C treatment, MDA increased by 41%–12%; and increased by 62%–41% at 0 °C. These results are consistent with previous studies (Liu and Ye, 2020; Panda and Gupta, 2024). Additionally, we found that MDA content continuously increased as the stress temperature decreased, with sensitive materials exhibiting a greater impact than cold-tolerant materials. Cold-tolerant materials showed almost no change in MDA content at 8 °C compared to the control, and the increase in MDA content at 4 °C and 0 °C was slower than that in sensitive materials. Sensitive materials consistently exhibited higher MDA levels than cold-tolerant ones. For example, during the germination stage, sensitive materials showed 20%–35% higher MDA than cold-tolerant ones at 8 °C, 23%–51% higher at 4 °C, and 27%–64% higher at 0 °C. This indicates that greater MDA variation in cold-tolerant materials under low-temperature stress correlates with reduced cold tolerance, demonstrating a negative relationship between MDA and cold tolerance. This finding aligns with the research by Hussain et al (Hussain and Li, 2023). The significantly higher MDA content in the cold-sensitive material compared to the tolerant material can be fundamentally attributed to an imbalance between the generation and scavenging of ROS. Under cold stress, the sensitive genotype fails to promptly activate its antioxidant defense system, leading to an over-accumulation of toxic free radicals. These excessive ROS directly attack the unsaturated fatty acids within the cell membranes, causing severe and rapid lipid peroxidation (Hasanuzzaman and Bhuyan, 2020; Ravi and Foyer, 2023). In contrast, the cold-tolerant material maintains higher membrane stability by efficiently clearing these radicals before they cause irreversible structural damage.

It is generally recognized that the content of Pro and soluble sugars positively correlates with plant resistance (Anokye and Lowor, 2021). Research findings indicate that following low-temperature stress exposure, both Pro and soluble sugar levels in cold-tolerant materials were higher than those in sensitive materials compared to the control group. During the germination stage, soluble sugar content in sensitive materials treated at 8 °C was 33.27%–40.25% lower than in cold-tolerant materials at 0–36 hours; At 4 °C treatment, sensitive materials exhibited 30.08%–38.15% lower levels; at 0 °C treatment, sensitive materials showed 31.11%–41.87% lower levels. This indicates plants regulate cold-induced cellular damage through rapid accumulation of both compounds, consistent with Yang et al.’s findings in rice under cold stress (Yang and Chen, 2023). The lower contents of Pro and soluble sugars in the cold-sensitive material are primarily due to its delayed and inefficient metabolic response. Cold-tolerant materials possess a ‘rapid-response’ genetic mechanism that quickly upregulates the biosynthesis of osmoprotectants to lower the intracellular freezing point and prevent cellular dehydration (Zuther and Buchel, 2004; O’Hara and Paul, 2013). However, in sensitive materials, severe cold stress rapidly disrupts normal cellular metabolism and energy allocation, thereby hindering the active synthesis and accumulation of these critical osmoregulatory substances (Wang and Liu, 2021). Additionally, the study found that cold-tolerant materials exhibited significantly higher levels of Pro and soluble sugars than sensitive materials. Furthermore, after stress cessation, the decline rates of Pro and soluble sugars differed between cold-tolerant and sensitive materials, with cold-tolerant materials decreasing more rapidly. This suggests that excessively high Pro and soluble sugar contents may adversely affect subsequent plant growth and development (Ji and Li, 2020).

The effects of low-temperature stress on SOD, POD, and CAT activities in cold-tolerant and sensitive materials revealed significant changes in all three activities. In our study, all three enzymes reached their peak activity at 0 hours post-cold stress. Taking POD as an example, POD activity at 0 hours under 8 °C, 4 °C, and 0 °C treatments was significantly higher than at 36 hours, increasing by 35.5%, 43.1%, and 46.5%, respectively. This finding is consistent with the results reported by Zhao et al. in cucumber (Zhao and Ye, 2016), where cold-tolerant materials exhibited a more rapid response than sensitive materials. SOD, POD, and CAT activities declined rapidly after the stress ended, demonstrating the faster response speed of the antioxidant enzyme system in cold-tolerant materials. This is consistent with studies by Kaisar, and others (Bhat and Mahajan, 2022). As the first line of defense in the ROS scavenging system, SOD exhibited higher oxygen ion conversion capacity in cold-tolerant materials compared to sensitive ones. The slow decline in sensitive materials indicates incomplete conversion of oxygen ions to H_2_O_2_ within plant tissues. POD and CAT, responsible for scavenging H_2_O_2_ produced by SOD, showed synchronous decreases with reduced H_2_O_2_ levels, demonstrating that cold-tolerant materials better maintain antioxidant system function after low-temperature stress. Specifically, the two materials exhibited distinct systemic responses to these enzymes: the tolerant material demonstrated a highly coordinated mechanism where SOD rapidly converted toxic superoxide radicals into H_2_O_2_, which was then safely detoxified by the synchronously elevated POD and CAT activities (Wei and Xu, 2020). Conversely, the sensitive material displayed a disjointed enzymatic response, where its inherently lower SOD activity failed to process the initial ROS burst, and the subsequent mismatch in POD and CAT activities ultimately led to the collapse of its antioxidant defense (Qin and Liu, 2024). In our study, the coordinated decline of all three enzymes after low-temperature stress suggests cold-tolerant materials can more effectively sustain antioxidant system activity. substrate depletion, suggesting cold-tolerant materials better maintain antioxidant system function under low-temperature stress. In our study, these three enzymes decreased coordinately after low-temperature stress cessation in cold-tolerant materials, whereas sensitive materials exhibited slow, delayed, and uneven decreases—consistent with Qin et al.’s findings in rapeseed (Qin and Liu, 2024).

Previous studies cold stress tests have focused on a single developmental stage. Our field screening at harvest combined comparisons of cold-tolerant extreme materials during both the seedling and germination stages. Pro showed significant or highly significant positive correlations with SOD and all other indicators, indicating that osmotic regulation and oxidative protection are simultaneously activated and mutually reinforcing physiological processes. Principal component analysis revealed that Pro and SOD activity measurements serve as core indicators for evaluating cold tolerance in peanut seedlings and young plants. Furthermore, the PCA results successfully integrated these multidimensional variables, highlighting their intrinsic functional correlations. In the PCA biplot, indicators such as Pro, SOD, and soluble sugars clustered closely together, exhibiting high positive loadings on the first principal component (PC1). This strong correlation visually and mathematically validates that osmotic regulation and antioxidant defense act synergistically rather than independently to confer cold tolerance. Meanwhile, the loading vector of MDA pointed in a distinct direction, confirming its negative correlation with the protective mechanisms. By condensing these complex physiological traits, PCA not only distinguishes the tolerant and sensitive genotypes clearly in spatial distribution but also emphasizes that a highly coordinated physiological network dictates peanut cold tolerance (de Freitas and Thomas, 2019).

Plants often exhibit significant differences in their sensitivity and response mechanisms to cold stress across different developmental stages. The germination stage, being one of the most vulnerable early phases of the plant life cycle, directly determines crop emergence and field establishment. Our study revealed that under extreme cold stress during the germination stage, the extreme cold-tolerant material px7 accumulated significantly lower levels of MDA compared to the sensitive material px30, while more rapidly initiating the accumulation of Pro and soluble sugars. This indicates that during the initial phases of seed imbibition and germination, px7 can rapidly stabilize cellular structures through highly efficient osmotic regulation, thereby ensuring normal radicle development. Conversely, px30 exhibited a delayed response and rapid decline in antioxidant enzyme (SOD, POD, CAT) activities during germination, leading to severe cell membrane lipid peroxidation. This physiologically explains its near 100% germination inhibition rate observed in the phenotypic screening.

As the plants advance to the seedling stage, the photosynthetic system in the leaves and more complex physiological metabolic networks become operational. Cold stress at this stage is highly prone to triggering a more intense ROS storm. Our comparative analysis showed that during the seedling stage, the cold-tolerant genotype px7 consistently maintained significantly higher activities of core antioxidant enzymes, such as SOD, and lower MDA damage levels than the sensitive genotype px30. This demonstrates that the genetic cold-tolerance mechanisms of px7 possess high continuity, stability, and systemic defense capacity when transitioning from the “heterotrophic” germination stage to the “autotrophic” seedling stage. In contrast, during the seedling stage of px30, not only were osmoprotectants (such as soluble sugars) rapidly depleted, but its enzymatic defense system also trended toward collapse, failing to effectively scavenge excess ROS and thereby accelerating the overall deterioration of the plant. Overall, px7 and px30 exhibited highly consistent physiological resistance differentiation during both the germination and seedling stages, which further corroborates the scientific validity and necessity of combining these two critical stages for the cross-screening of cold tolerance in large-scale peanut germplasm evaluations.

## 5 Conclusion

Following exposure to low-temperature stress, peanut plants undergo a series of internal physiological changes, the extent of which largely depends on the cold tolerance of the genotype. This study screened an extremely cold-tolerant material and an extremely cold-sensitive material from field trials. Physiological assessments conducted during the germination and seedling stages revealed that the cold-tolerant genotype exhibited a faster decline in MDA content during the recovery phase, indicating that the lipid membranes of sensitive genotypes face greater difficulty in repairing cold damage. Conversely, osmoprotectants (i.e., Pro and soluble sugars) rapidly declined in the sensitive material while remaining elevated (with a slower decline) in the cold-tolerant material, facilitating more sustained and effective ROS scavenging. Furthermore, antioxidant enzyme activities consistently remained lower in the sensitive material than in the cold-tolerant material across both developmental stages. PCA collectively indicated that measuring Pro content and SOD activity serve as core physiological indicators for evaluating peanut seedling cold tolerance. Although this study primarily elucidates differences in cold tolerance mechanisms at physiological and biochemical levels, the screened px7 and px30 genotypes not only exhibit stable cold tolerance differences across both developmental stages but also possess unique genetic backgrounds. This makes them valuable candidate germplasm resources for future exploration of peanut cold tolerance molecular mechanisms (e.g., transcriptomic mining of key cold-response genes like CBF or ICE1) and quantitative trait locus (QTL) mapping.

## Data Availability

The original contributions presented in the study are included in the article/**Supplementary Material**, further inquiries can be directed to the corresponding author/s.
